# Developing faculty leadership from ‘within’: a 12-year reflection from an internal faculty leadership development program of an academic health sciences center

**DOI:** 10.1080/10872981.2019.1567239

**Published:** 2019-02-04

**Authors:** Janice Y. Tsoh, Anda K. Kuo, John W. Barr, Laney Whitcanack, Irené Merry, Brian K. Alldredge, Amin N. Azzam

**Affiliations:** a Department of Psychiatry, University of California San Francisco, San Francisco, CA, USA; b Department of Pediatrics, University of California San Francisco, San Francisco, CA, USA; c High Desert Museum, Bend, OR, USA; d Coro Northern California, San Francisco, CA, USA; e Office of Vice Provost, Academic Affairs and Faculty Development, University of California San Francisco, San Francisco, CA, USA; f Departments of Clinical Pharmacy and Neurology, University of California San Francisco, San Francisco, California, USA

**Keywords:** Leadership development, faculty development, health sciences center

## Abstract

Most academic health sciences centers offer faculty leadership development programs (LDPs); however, the outcomes of LDPs are largely unknown. This article describes perspectives from our 12-year experience cultivating a formal faculty LDP within an academic health center and longitudinal outcomes of our LDP. Responding to faculty concerns from University of California San Francisco’s (UCSF) 2001 Faculty Climate Survey, UCSF established the UCSF-Coro Faculty Leadership Collaborative (FLC) in 2005. The FLC focused on building leadership skills using a cohort-based, experiential, interactive and collaborative learning approach. From 2005 to 2012, FLC has conducted training for 136 graduates over 7 cohorts with 97.6% completion rate. FLC faculty participants included 64% women and 13% underrepresented minority (URM). The proportions of graduates attaining leadership positions within UCSF such as deans or department chairs among all, URM, and women URM graduates were 9.6%, 33.3% and 45.5%, respectively. A 2013 online survey assessed 2005–2012 graduates’ perceived impacts from 8 months to 8 years after program completion and showed 91.7% of survey respondents felt the program both increased their understanding of UCSF as an organization and demonstrated the University’s commitment to foster faculty development. Qualitative results indicated that graduates perceived benefits at individual, interpersonal, and organizational levels. Though we did not directly assess impact on faculty recruitment and retention, the findings to date support cohort-based experiential learning in faculty leadership training development.

## Introduction

With increasing urgency, academic health sciences centers (AHCs) and health professional organizations are recognizing the need to foster the core competency of leadership, within their workforces and organizational cultures [1–3]. Indeed, among the 94 AHCs in Canada and the United States that responded to a national survey from the Study of Leadership Development Programs at North American Academic Health Centers in 2015 [4], 99% of the responding AHCs provided some form of leadership development training such as informal single workshops, formal internal faculty leadership development programs (LDPs), or formal external LDPs. Over half (65%) of these AHCs reported offering at least one formal LDP. While research intense institutions were more likely to have LDPs; 42% of AHCs that did not offer a formal LDP cited lack of resources as a significant barrier. A majority of the AHCs (73%) sent participants to external LDPs rather than cultivating an LDP within their own institution.

In spite of increasing availability of leadership development opportunities, LDPs often fail to demonstrate efficacy in creating leaders [5–10]. In part, this is because of minimal program assessment. For example, while AHC LDPs obtain participant satisfaction data, less than 50% assess other outcomes, such as actual learning, behavioral change, or post program individual achievement [4]. In addition, AHC LDPs most commonly deliver content in a traditional classroom format. This ignores best practices for the development of leaders, which include experiential application of skills, assessment of individual personal growth, and reflection on action [5–11].

This article describes experience cultivating a LDP within an AHC, the University of California San Francisco (UCSF) in full partnership with Coro Northern California (Coro), a nonprofit organization focused on leadership development that uses a cohort-based experiential learning approach [12]. Between 2005 and 2017, the 12-year UCSF-Coro Faculty Leadership Collaborative (FLC) had over 180 graduates. We conducted an online survey in 2013 with graduates from 2005 to 2012 that assessed participants’ perceived downstream behavioral change, including both individual growth and impact on institutional climate. We also reported program completion rate and proportions of graduates from the 2005 to 2012 cohorts attaining leadership positions within the institution (UCSF).

The authors are members of UCSF faculty leadership, Coro executive leadership, FLC staff administrators, and FLC graduates who have first-hand experience in various phases of the FLC implementation and outcome evaluation. The objective of this paper is to reflect 12 years of perspectives on cultivating a LDP within an AHC by describing the history, implementation, and longitudinal outcomes of FLC.

## Methods

### History of the UCSF-Coro FLC

In 2001–02, the UCSF Chancellor commissioned a survey on the climate at UCSF for faculty, particularly women faculty. A total of 1,057 faculty responded (60% participation rate). The survey findings revealed that women faculty indicated significantly more dissatisfaction than men faculty with numerous areas including job demands, income, and leadership opportunities [13]. In 2003, a Task Force convened to review the survey results and made 10 recommendations, one of which included development of leadership skills and opportunities, especially for women faculty [14]. This led to the formation of the Chancellor’s Council on Faculty Life (CCFL), chaired by the Vice Provost of Academic Affairs. In 2004–05, the UCSF Office of Academic Affairs issued a Request for Proposals (RFP) for a leadership training provider. Two organizations responded to the RFP: Coro Northern California and one internal group. CCFL made the decision to engage with Coro who might bring a different perspective on leadership development from their predominant work which was outside of academic institutions. Coro Northern California (https://coronorcal.org), a nonpartisan nonprofit organization, has offered leadership training program since 1947. Coro centers across the United States have graduated more than 11,000 leaders from their various programs. Coro Northern California has worked with more than 700 faculty and administrators affiliated with the University of California campuses since 2006 in various LDPs. Additionally, 1,600 high-school students and young professionals have graduated from Coro programs. The UCSF collaboration with Coro started in 2005.

### Training philosophy

The UCSF-Coro FLC is a program administered within UCSF by Coro. Coro experiential training model was developed in the 1940s. The training model shares similar concepts of Kolb’s experiential learning model [15], which was refined from Dewey’s educational principles emphasizing concepts such as experience, experiment, observation and reflection, and purposeful learning [16]. Coro’s design of curriculum and learning activities encourages participants to accept new tools introduced in the training, apply the tools, and to then adapt the tools, as needed, based on one’s experiences and relevancy after a process of reflection. Coro uses an array of tools to focus on developing six core leadership competencies: 1) self-awareness, 2) critical thinking, 3) effective communication, 4) inclusion, 5) collaboration, and 6) empowered professionalism (confidence to represent oneself consistently in many different environments), rather than following a specific leadership model.

The FLC aims to facilitate networking among its participants within the AHC through a cohort-based model of training. Coro trainers conduct FLC training within the AHC, with the goal of catalyzing individual and collective change to benefit both the participants and the broader UCSF community. The training centers on self-reflection, critical evaluation, and basic leadership skills (e.g., how to run a meeting, how to take and give feedback, public speaking, etc.). FLC includes meetings with selected UCSF leaders. Through preparing and conducting group-based in-depth interviews with various UCSF leaders, participants learn to apply Coro tools to critically analyze group processes and learning outcomes.

### Implementation of UCSF-Coro FLC

FLC is inclusive of UCSF’s four schools of Dentistry, Medicine, Nursing, and Pharmacy. UCSF departments select faculty members for FLC who they believe will provide leadership for their colleagues, the departments, and the medical center community. Nominations are solicited through the Office of the UCSF Vice Provost of Academic Affairs. Local-level leaders, such as department chairs, division chiefs or campus site specific hospital administration chiefs of staff, are encouraged to nominate faculty members whom they believe would provide leadership for their colleagues, the departments, and the medical center community. All faculty appointed at 50% or greater paid effort and who have been in a UCSF faculty position for more than 4 years are encouraged to apply. A statement of support from the applicant’s department chair is required, and the applicant must attest that the chair supports the time commitment required to fully participant in the program.

Selection amongst the applicant pool is determined by a selection committee, which is composed of campus administrators and past FLC participants. Applicants are rated upon: personal initiative; ability to work well in diverse groups; desire to contribute to the campus community; intellectual curiosity; and evidence of leadership potential or experience from the applicants’ curriculum vitae, personal statement and chair’s letter of support. When selecting applicants for admission to the program, the committee made a conscious effort to address issues of equity and inclusion (e.g., by gender, URM status, school, and department).

Cohorts of up to 16 faculty members are given release time from their clinical/research/administrative responsibilities to complete a 10-session (75 program hours across approximately 20 weeks) leadership program. The release time is not based on salary scale, but is protected time to attend all FLC-related activities. Participants’ release time is negotiated at the local department or division level. An example of the 10-session program training contents is provided in Appendix 1.

### Evaluation of UCSF-Coro FLC: study design

UCSF-Coro is an ongoing program. Since FLC inception in 2005, there has been 10 cohorts (through 2017) of FLC graduates. In 2013, we conducted an online survey of the graduates from the 2005–2012 FLC cohorts to assess the longitudinal impact of program participation. The evaluation FLC was limited to the first 7 cohorts of FLC graduates enrolled between 2005 and 2012 and was assessed in two primary aspects: (1) leadership attainment within UCSF among 2005–2012 FLC graduates and (2) FLC graduates’ perceived impacts of FLC using an online survey conducted in 2013.

### Survey instrument

The survey was developed by a team of UCSF faculty, graduates from the FLC, and Coro employees familiar with the FLC and other Coro LDPs. The final version was vetted and approved by the UCSF CCFL. We used a mixed-methods design incorporating quantitative and qualitative items: 13 demographic items (e.g., age, gender, academic rank, school, program, etc.); 18 Likert scale and multiple-choice questions that asked perceived impacts of program participation on various dimensions related to program goals (confidence building, enhanced attitudes towards one’s role, networking and interactions, campus climate, faculty recruitment or retention); and 16 open-ended questions designed to capture respondents’ perspectives in their own voices for the above-mentioned dimensions and to solicit any additional experience with the FLC. The survey is included in Appendix 2. Survey procedures were approved by UCSF’s institutional review board.

### Survey participant recruitment

In August 2013, out of the 136 graduates of the 2005–2012 FLC cohorts, 131 had a valid email address and were invited to participate via an email solicitation, which included 19 (14.5%) who were no longer at UCSF. Two reminder emails spaced 2-weeks apart encouraged non-respondents. By close of data collection in October 2013, 72 respondents (55% response rate) began or completed the survey. Respondents included 12 (17%) who were not at UCSF.

### Leadership attainment outcome

We defined leadership attainment as FLC graduates who attained a leadership position at UCSF, defined as Dean (including Vice and Associate Dean positions), Department Chair, and Director of Organized Research Unit. This outcome definition has been used to obtain funding allocation within the institution to support the continued provision of leadership development and training.

### Data analysis

Using IBM SPSS Statistics 24.0 (IBM Corporation, New York) for quantitative data analysis, we computed means, standard deviations, and percentages. The 4-point Likert scale items were dichotomized to ‘strong disagree/disagree’ versus ‘strongly agree/agree’ due to bimodal distributions observed of the items. For open-ended qualitative items, we used Atlas.ti 8.0 (ATLAS.ti GmbH, Germany) to organize and code the data. Author team members independently reviewed the qualitative responses, developed initial codes, and formulated candidate themes. Through iterative group reviews and discussions, we refined and generated a final set of themes and subthemes. We integrated quantitative findings with emergent qualitative themes. For leadership attainment, frequency counts and percentages were computed for the entire sample of 2005–2012, and by the following graduate categories of focus: women faculty, URM, and women URM faculty. URM was defined as ethnicities and racial groups considered to be URMs in health sciences, which included African American/Black, Filipino or Hmong or Vietnamese Asians, Hispanic/Latino, Native American/Alaskan Native, or Native Hawaiian/Other Pacific Islander, or two or more races with one or more from the URM categories [17].

## Results

### FLC participant characteristics

Since the inception of UCSF-Coro FLC in 2005 through 2012, the program conducted training with 7 cohorts (Table 1). A total of 136 graduates included 65.4% women and 13.2% URM (defined as ethnicities considered to be URMs in health sciences). Among URM graduates, 61.1% were women. The program provided training to faculty at all ranks. FLC completion rate is 97.8%. FLC graduates included faculty from 33 departments, including a majority from the school of Medicine (79.4%), and the remaining were from schools of Dentistry (10.3%), Nursing (5.9%), and Pharmacy (4.4%). As the size of each school has varied slightly over time, the distribution by school of the FLC graduates were comparable to the median proportions of the faculty across the schools during the same period of time: Medicine (81.6%), Dentistry (7.4%), Nursing (5.6%), and Pharmacy (4.0%).

**Table 1.** UCSF-Coro Faculty Leadership Collaborative participant characteristics for the entire graduate cohort of 2005–2012 and respondents of the 2013 graduates survey.

UCSF-Coro Faculty Leadership Collaborative Cohorts	2005–2012 FLC graduates (*n* = 136) *n* (%)	2013 FLC graduates survey respondents (*n* = 72) *n* (%)
Gender Female Male	89 (64.4) 47 (34.6)	47 (66.2) 25 (33.8)
Race/ethnicity Non-underrepresented minority (Non-URM: White or Asian) URM (e.g., Black, Latinos)	118 (86.8) 18 (13.2)	59 (83.1) 12 (16.9)
Program cohorts (year) 2005–2006 2006–2007 2008 2010 2011 2012	32 (17.7) 31 (17.1) 31 (17.1) 16 (8.8) 10 (5.5) 16 (8.8)	18 (25.4) 11 (15.5) 13 (18.3) 13 (18.3) 7 (9.9) 9 (12.7)
School (primary appointment at start of the program) Dentistry Medicine Nursing Pharmacy	14 (10.3) 108 (79.4) 8 (5.9) 6 (4.4)	5 (7.0) 60 (84.5) 4 (5.6) 2 (2.8)
Academic rank at* Assistant professor Associate professor Full professor	*FLC enrollment 35 (25.7) 61 (44.9) 40 (29.4)	*Survey completion 3 (4.3) 15 (21.4) 52 (74.3)
Degrees (not mutually exclusive) MD, DMD PhD, DPH Other clinical degrees (e.g., DDS, PharmD, PsychD, RN, PNP, PT)	76 (55.9) 58 (42.6) 21 (15.4)	Not assessed

Percentages were computed out of the non-missing responses; mutually exclusive categories may not be summed up as 100.0% due to rounding.

### Leadership attainment of FLC participants

**Figure 1.**
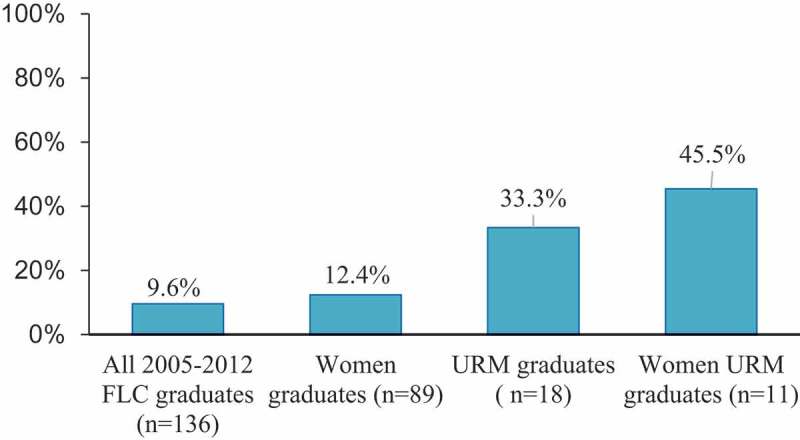
Percentages by FLC 2005–2012 graduate subgroups who attained leadership position within UCSF by year 2017.

As of 2017, 9.6% (*n* = 13) of the graduates attained leadership position at UCSF, defined as Dean (including Vice and Associate Dean positions), Department Chair, and Director of Organized Research Unit. The proportions of FLC graduates attaining leadership position varied greatly by schools: Medicine (5 of 108; 4.6%), Dentistry (4 of 14; 28.6%), Nursing (2 of 8; 25%), and Pharmacy (2 of 6; 33.3%). Among the 13 FLC graduate leaders, 11 were women faculty and 6 were URM faculty (including 5 women and 1 man). Figure 1 depicts the percentages of leadership attainments by selected FLC graduate groups including the entire sample, women, URM and woman URM faculty. In comparison to the 2017 UCSF faculty body, which included 50% women and 8% URM, the data provide evidence of FLC’s effectiveness in recruiting both women and URM faculty, and also in supporting women faculty in attaining leadership within our AHC.

**Figure:**
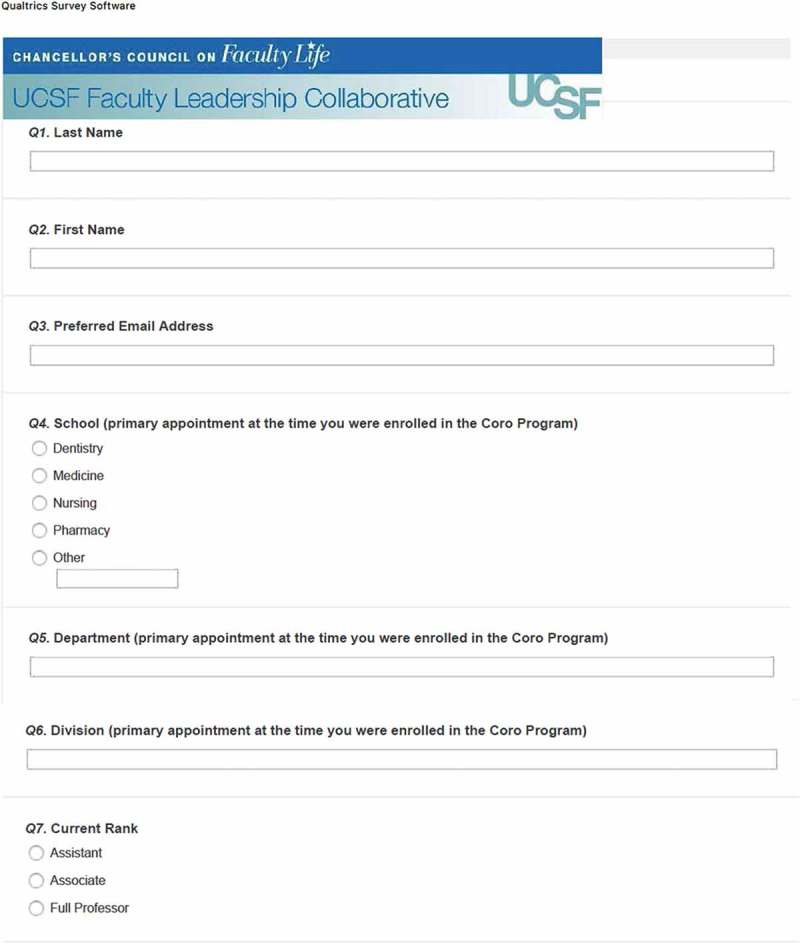

**Figure:**
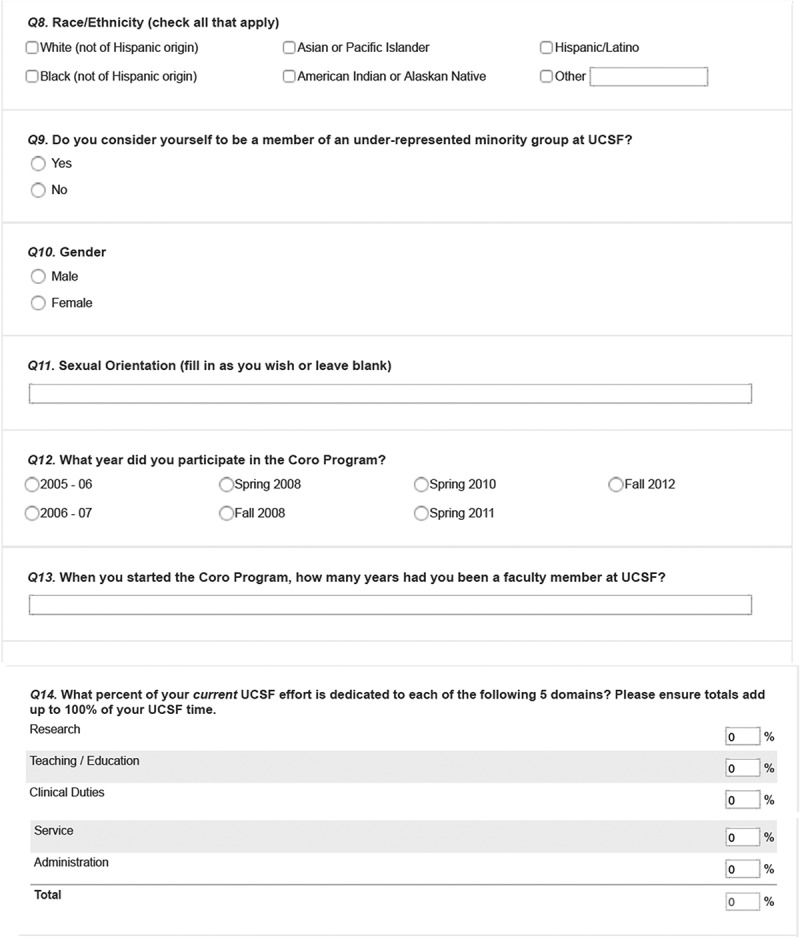

**Figure:**
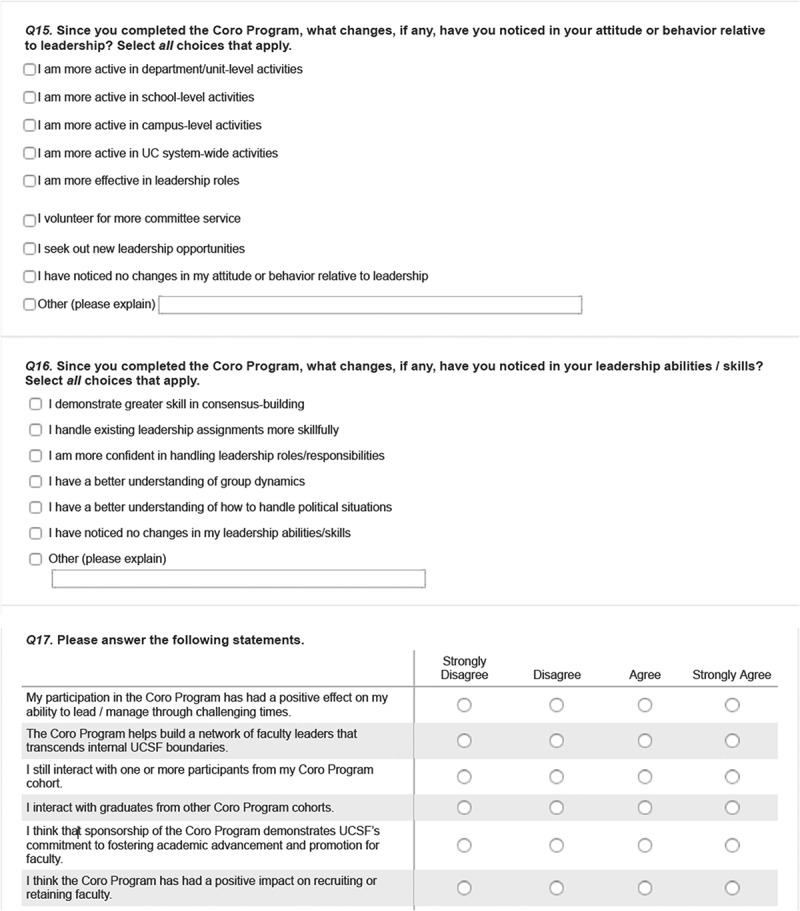

**Figure:**
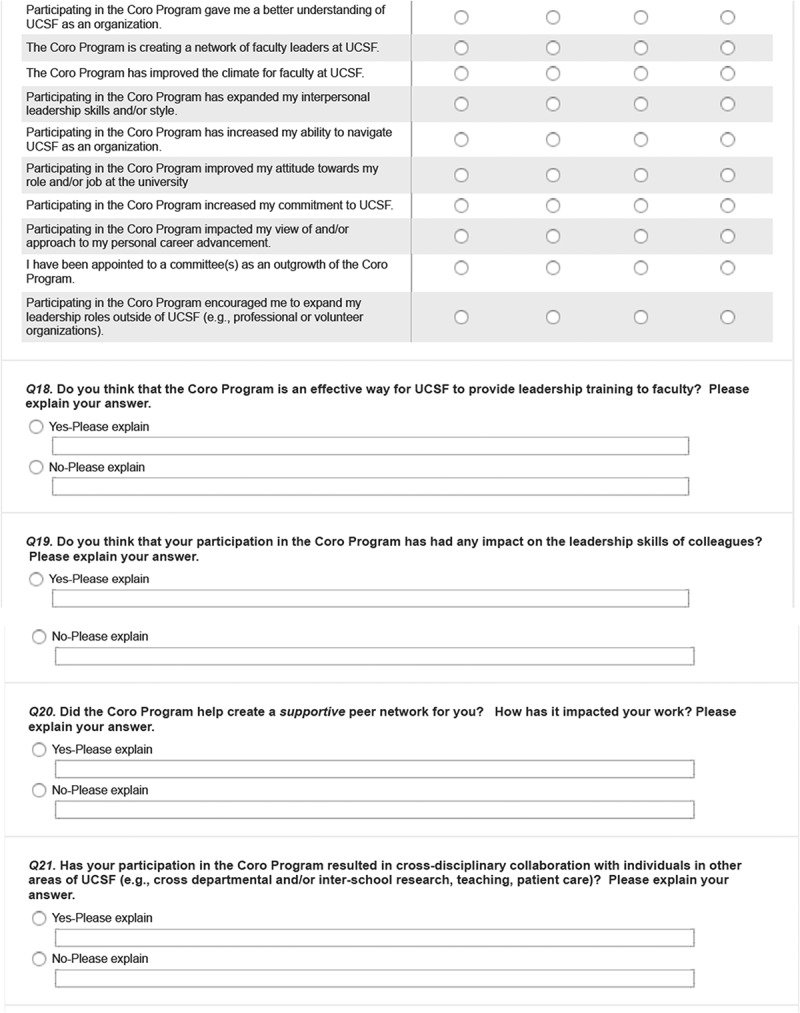

**Figure:**
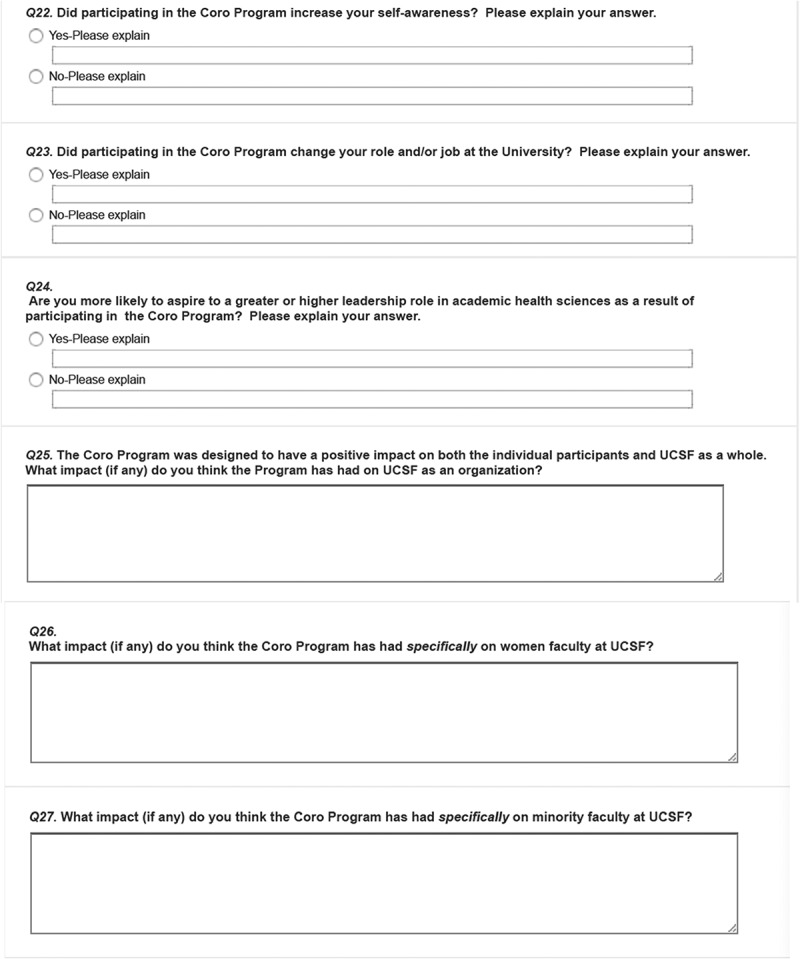

**Figure:**
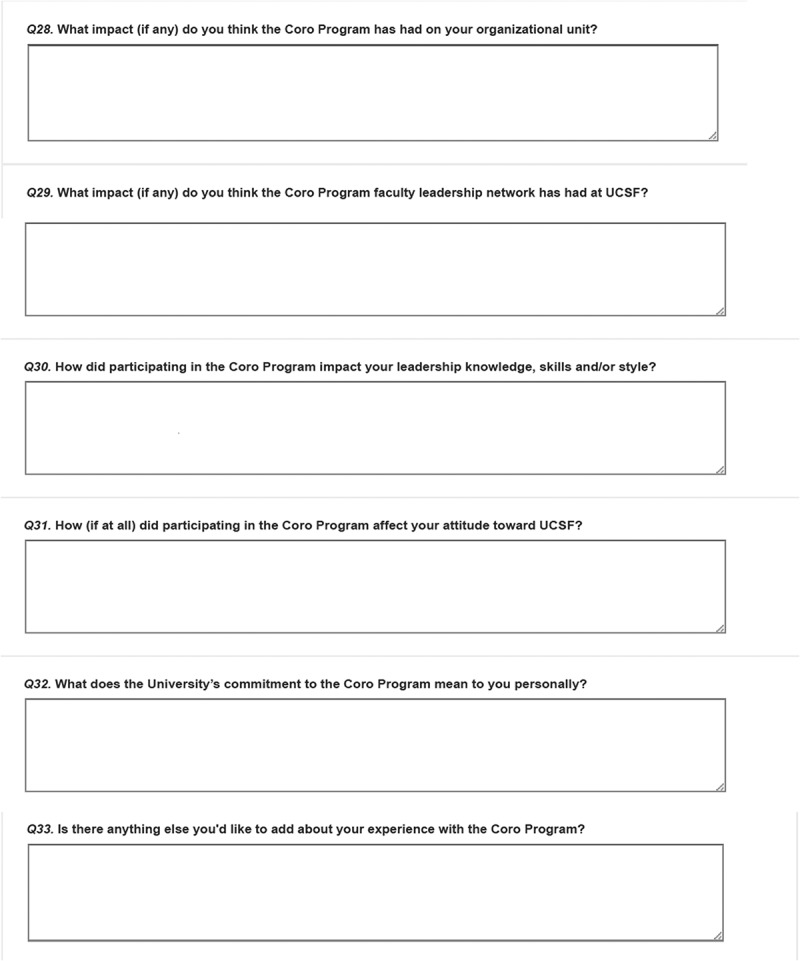

### Sample characteristics of survey respondents

Table 1 shows the characteristics of the 72 survey respondents. Overall response rate was 55.0%, ranging from 35.5% to 81.3% across cohorts. The sample included 66% women. Respondents’ self-identified race and ethnicity included 64.8% White, 18.3% Asian, 9.9% Black, and 7.0% Latino. When starting the FLC, the mean years a participant had been a UCSF faculty member was 10.8 years (SD = 6.8; range 1–27 years). One-third reported research as their primary faculty role, other primary roles included clinical service (18.8%), administration (18.8%), teaching (17.4%), or multiple primary roles (11.1%).

### Perceived impacts from FLC from survey respondents

The FLC, as perceived by the program participants 12 months to 8 years after program completion, has generated positive and sustaining impacts at multiple levels. Based on thematic analyses, the levels of impacts were categorized to (1) individual, (2) interpersonal, and (3) organizational levels.

#### Theme 1: individual level impacts

At an individual level, the program fostered development of leadership skills in conflict resolution, team management, and giving and receiving feedback. Since completion of the FLC, a majority indicated noticeable changes in leadership skills (98.6%), and attitudes or behaviors related to leadership (91.7%). Specifically, the most commonly endorsed individual changes include a positive effect on ability to lead or manage through challenging times (93.1%), increased confidence in handling leadership roles or responsibilities (80.6%), and increased effectiveness in leadership roles (77.8%). Post completion of the FLC, while only 29% indicated volunteering more for committee service, 62.5% reported seeking out new leadership opportunities, and 61.1% were appointed to one or more committees as an outgrowth of the program. More women than men agreed their program participation encouraged them to expand their leadership roles in professional or volunteer organizations outside of UCSF (72.7% vs. 47.8%; χ^2^(1) = 4.1, *p* = 0.04).

Qualitative responses supported impacts of participation in the FLC on personal growth, particularly in increasing self-awareness, confidence, and aspiration. For example, in response to how participating in the FLC has changed one’s self-awareness, one participant stated, ‘It made me much more aware as an effective leader using my strengths and learning to optimize my weaknesses.’ Another participant indicated, ‘… [it] made me more confident as a leader and yet more willing to listen to others and give credit to them for their ideas.’

#### Theme 2: impacts at interpersonal level

Graduates described how the program led to new collaborations. Most (90%) reported increased interpersonal leadership skills and 85% indicated their participation helped build a network of faculty leaders transcending internal departmental boundaries. Two-thirds (67%) said their participation gave them better understanding of group dynamics. Post FLC completion, 69% and 50% said they continued to interact with participants from their or other cohorts, respectively.

The qualitative data from respondents described the FLC as a means to provide support, connectedness, empowerment, and formal mentorship, particularly for women and minority faculty. One graduate indicated, ‘Collaboration between participants is the real “gem” behind the program. I realized how diverse UCSF really is….’ Some graduates described experiencing increased collaboration across and increased cohesion within departments or units. One graduate commented, ‘…helping me be a better leader within my unit, and helping my unit by serving as a bridge to other people across UCSF.’ Another graduate commented, ‘I feel more engaged with the university as a whole, and more interested in collaborating with others outside of my department.’

#### Theme 3: impacts at organizational level

Most respondents (91.7%) agreed that the sponsorship of the FLC demonstrated the University’s commitment to foster faculty development. A majority (91.7%) said the program had increased their understanding of UCSF as an organization; 79.2% credited the FLC to an increased ability to navigate UCSF as an organization. A majority (76.4%) said they developed an improved attitude towards their role and/or job at the university. Two-thirds (66.2%) perceived a positive impact on recruiting or retaining faculty or agreed that participating in FLC increased their commitment to UCSF. More Full Professors agreed to the statement that the program improved the climate for UCSF than Associate or Assistant Professors (78.8% vs. 45.0%; χ^2^(1) = 7.8, *p* = 0.005). One graduate commented, ‘It is an important symbol to me, personally, of the university’s interest in “growing its own” and, in particular, of this public institution’s continued interest in trying to assure that its leaders look like the public the university serves.’

## Discussion

We report on longitudinal outcomes associated with a faculty LDP at one AHC and run by a nonprofit experiential learning organization. Longitudinal outcomes are rarely reported in the faculty development literature, yet our study includes self-reported outcomes as long as 8 years post-completion. Our FLC provides faculty participants with a strong experiential process, in particular cultivating and strengthening a culture of a collaborative network. Tools and experience gained through the 10-session program increase the sense of community, bringing people working in silos together. In particular, women and URM faculty are overrepresented among program graduates as compared with their representation among the at-large faculty, and in the number and diversity of faculty leadership program graduates who have gone on to school-level and department-level leadership positions. Although the impact on faculty recruitment and retention was not directly/precisely assessed, a majority of faculty positively endorsed the investment made by the University in faculty development through sustained commitment to leadership training. There is also high satisfaction and self-reported impact on individual, interpersonal, and institutional levels.

While subsequent iterations continue to evolve both content and structure, more can be done to sustain these multi-level impacts. Strategies might include (1) additional support beyond the program to maintain cross-institutional connections and peer-support, (2) reinforcement and further skill-building (e.g., booster sessions to bring graduated cohorts together), and (3) support for graduates in accelerating transitions to new leadership positions (e.g., ‘follow-on experiences’ to sustain or reinforce new skills and move the graduates more swiftly towards efficacy in various leadership roles).

As lack of funding is frequently cited as a barrier to LDPs at AHCs [4], we offer the history of funding of the UCSF FLC as one example of an evolving model that led to sustained institutional fiscal commitment. Since 2005, CCFL has twice received 5-year funding allocations that support the provision of leadership development and training. Communications to campus leadership to justify continued funding of leadership training have focused on 1) visibility and popularity of the program among the UCSF faculty (i.e., high demand); 2) number of faculty trained in 10-session and shorter versions of the leadership program; and 3) diversity among those faculty who have received leadership training assessed by gender, school, department, faculty series, and URM status. In 2016, funding for CCFL and associated leadership training was institutionalized (e.g., no longer subject to allocation review every 5 years).

Our survey study has several limitations. We did not measure or verify respondents’ self-reported behavior or attitude change and did not collect details on career trajectories of the graduates other than their subjective opinions of the program. We have limited our analysis by only reporting leaders at the dean, chair or director level within UCSF, and did not have the attainment of leadership positions of past graduates who have moved to other institutions, which may likely underestimate the potential impact/effect size of participation in the FLC. Another limitation is the lack of a randomly matched cohort of faculty who did not attend the FLC. While it would be complex to retrospectively define matched cohorts over a longitudinal decade long time frame, that comparison group would significantly strengthen these findings. An additional limitation is the nomination process for inclusion in the FLC. Department Chairs, Deans, and other nominating leaders would have selected names based on subjective perceptions of leadership potential. The nomination process may vary by schools and have contributed to the big differences in percentages of leadership attainment of FLC graduates across schools (5% among School of Medicine graduates to 25% among School of Nursing graduates). This may similarly have systematically biased the enrollees towards a non-representative sample of faculty who would have succeeded even without participating in the FLC. Lastly, as there are always multiple sources of influence for leadership development, we cannot directly attribute the positive outcomes or individual career advancement exclusively to the FLC. Nevertheless, the positive outcomes demonstrated by successful outreach to women and URM faculty and the positive feedback from FLC graduates have facilitated and maintained the ongoing FLC collaboration between UCSF and Coro for more than 12 years.

## Conclusion

For 12 years, the UCSF-Coro FLC successfully has reached a diverse faculty audience and created a faculty leadership network. The observational outcome is encouraging, especially among URM and among woman URM faculty with one-third to nearly half of these faculty graduates attaining leadership positions at UCSF. At least by self-report of FLC graduates, positive impacts from FLC appear longitudinally sustained as many as 8 years post-completion. The positive impacts spanned across multiple levels, such as personal growth, enhancement in leadership attitudes, increased interpersonal leadership skills and collaborations, increased understanding of their home institute, and perceived commitment of faculty development from the institution. Our 12-year reflection from the UCSF-Coro FLC, an internal faculty LDP in an AHC utilizing cohort-based experiential learning supports the feasibility for developing faculty leadership from ‘within.’ This approach has yielded sustainable objective and self-reported positive outcomes at individual, interpersonal, and organization levels. Future research might include assessment of the impact of the FLC on faculty retention, advancement, assumptions of leadership roles, and climate perceptions.

## Previous presentation

A preliminary version of findings was presented at 2017 Western Group on Educational Affairs (WGEA) Regional Meeting, Salt Lake City, 26 February 2017.

## Appendices

### Appendix 1. UCSF-Coro leadership collaborative program module overview

**Module 1: UCSF Campus Exploration**
  - Key result: Greater familiarity with UCSF through a logic study of a campus unit, experience with each other, their group dynamic, the Coro process and members of the UCSF Community.
  - Competency: Understanding of a campus institution, group dynamics (e.g., how the group gathers and communicates information, assesses leadership, and achieves goals).
**Module 2: Skills-based Day on Self and Group Awareness**
  - Key result: Greater self and group awareness, a greater sense of trust among participants.
  - Competency: Outcome-based leadership practices, interactive awareness, and inquiry as an essential element of leadership and change.
**Module 3: Skills-based Day on Balancing Inquiry and Advocacy**
  - Key result: A baseline understanding of what advocacy and inquiry looks/feels like, and an increased ability to be aware of when it is being used.
  - Competency: Advocacy and inquiry; formulating questions; analyzing responses for facts and opinions.
**Module 4: Organizational Awareness and Leadership**
  - Key result: Increased understanding of the structure and function of organizations by examining UCSF and/or schools and departments; identify opportunities for improving organizational performance through analyses of structure and staffing.
  - Competency: Introduce and apply tools for organizational analysis, fact based inquiry and adaptive and technical leadership.
**Module 5: Strategic Messaging at UCSF**
  - Key result: Greater awareness of how to shape key messages, debates and policy through a basic understanding of strategic communications. Increased awareness of fellow participants’ work projects through active listening.
  - Competency: Public speaking skills, active listening, interviewing, elevator pitch.
**Module 6: Skills-based Day on Listening and Feedback**
  - Key result: Understanding of how feedback and listening are essential elements of leadership, interactive awareness, and creating change.
  - Competency: Feedback, listening, peer coaching.
**Module 7: Skills-based Day on Listening and Feedback with In-depth Peer-to-peer Practice**
  - Key result: Increased ability to give and receive feedback in a group and individual context. Greater awareness of how individual participants affect each other and with this knowledge, a greater ability to better achieve both individual and group goals.
  - Competency: Giving and receiving constructive feedback.
**Module 8: Skills-based Day on Conflict Resolution & Negotiation**
  - Key result: Increased awareness of individual strengths and weaknesses in negotiations and conflict resolution, and an understanding of negotiations and conflict resolution principles and practices.
  - Competency: Negotiations and Conflict resolution principles and practices, skills for managing emotional situations.
**Module 9: Strategic Planning and Implementation**
  - Key result: Increased understanding of organizational systems and the strategic planning process at UCSF-successes and challenges-through interview of university leader(s) involved.
  - Competency: Systems thinking: awareness of the systems in which participants are, and the role they play in them, strategic and/or backwards planning.
**Module 10: Wrap-Up & Closure**
  - Key result: Understanding of how participants can and want to sustain relationships beyond Coro to create opportunities for ongoing coaching, resources and consulting. Greater familiarity with Coro’s trainer and other programs and services, and a sense of closure regarding the formal Coro experience.
  - Competency: collaborative group governance
